# Supplementary material for: Conditional chemoconnectomics (cCCTomics) as a strategy for efficient and conditional targeting of chemical transmission
Source: eLife. 2024 Apr 30;12:RP91927. doi: 10.7554/eLife.91927 (PMC11060718; doi:10.7554/eLife.91927)
Supplement: Figure 3—source data 3. [file elife-91927-fig3-data3.zip › Figure 3 source data3 part 1.zip!/Figure 3-Source data 3-part1/1175-9-CRISPResso_Report_rtnd62ko.zip!/CRISPResso_on_1175_10/9.Alleles_frequency_table_around_sgRNA_gtaggccacgcagtagacca.pdf]

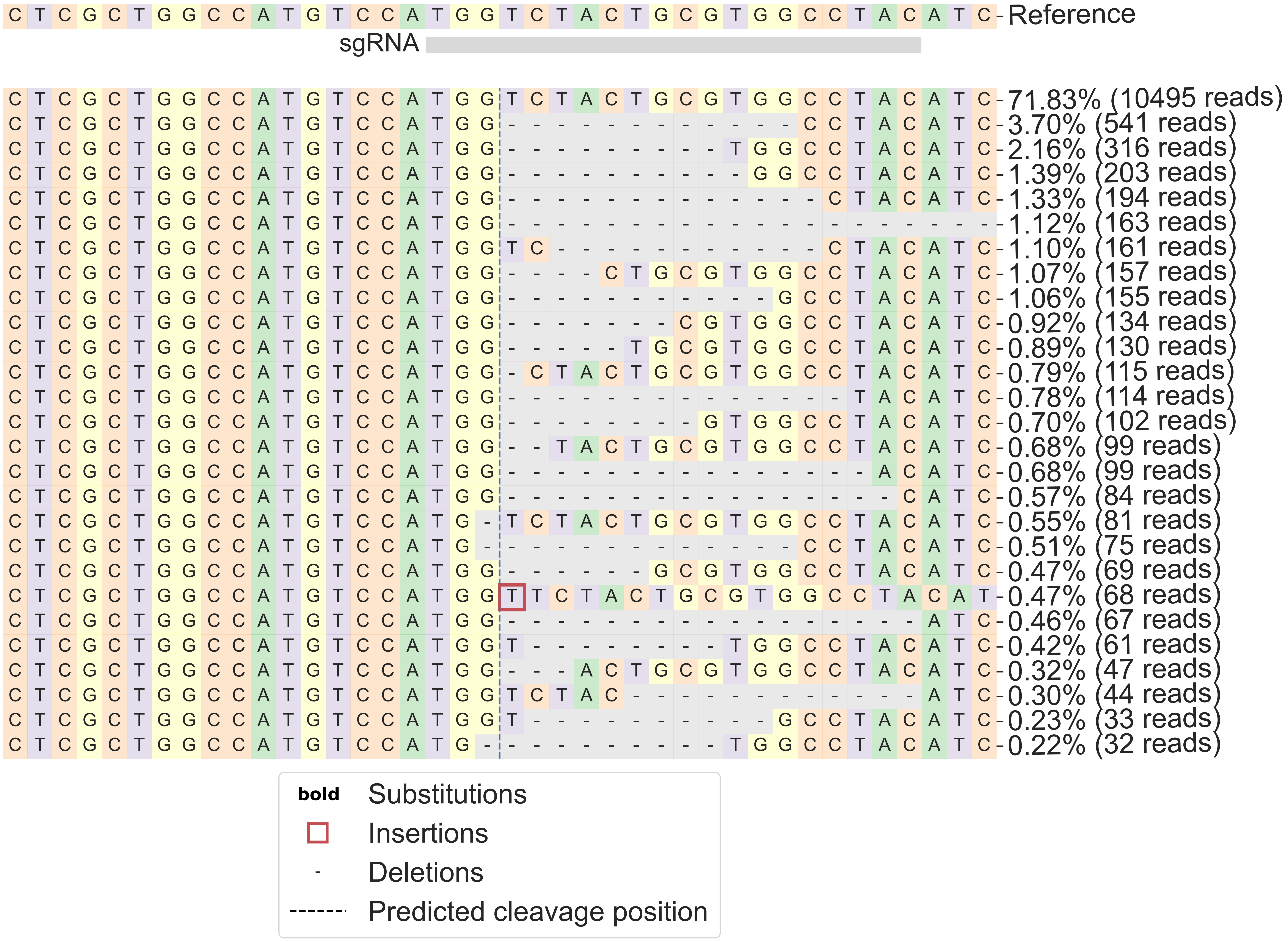
C T C G C T G G C C A T G T C C A T G G T C T A C T G C G T G G C C T A C A T C - Reference
sgRNA
71.83% (10495 reads)
3.70% (541 reads)
2.16% (316 reads)
1.39% (203 reads)
1.33% (194 reads)
1.12% (163 reads)
1.10% (161 reads)
1.07% (157 reads)
1.06% (155 reads)
0.92% (134 reads)
0.89% (130 reads)
0.79% (115 reads)
0.78% (114 reads)
0.70% (102 reads)
0.68% (99 reads)
0.68% (99 reads)
0.57% (84 reads)
0.55% (81 reads)
0.51% (75 reads)
0.47% (69 reads)
0.47% (68 reads)
0.46% (67 reads)
0.42% (61 reads)
0.32% (47 reads)
0.30% (44 reads)
0.23% (33 reads)
0.22% (32 reads)
bold Substitutions
Insertions
Deletions
Predicted cleavage position
